# Development of an Aptamer-Based QCM-D Biosensor for the Detection of Thrombin Using Supported Lipid Bilayers as Surface Functionalization

**DOI:** 10.3390/bios14060270

**Published:** 2024-05-25

**Authors:** Anne Görner, Leyla Franz, Tuba Çanak-Ipek, Meltem Avci-Adali, Anna-Kristina Marel

**Affiliations:** 1 Department of Food Technology and Bioprocess Engineering, Max Rubner-Institut, Federal Research Institute of Nutrition and Food, 76131 Karlsruhe, Germany; anne.goerner@mri.bund.de (A.G.); leyla.franz@mri.bund.de (L.F.); 2 Department of Thoracic and Cardiovascular Surgery, University Hospital Tübingen, 72076 Tübingen, Germany; tuba.canak-ipek@uni-tuebingen.de (T.Ç.-I.); meltem.avci-adali@uni-tuebingen.de (M.A.-A.)

**Keywords:** aptasensor, lipid bilayer, reconstitution, QCM-D, thrombin, serum

## Abstract

Biosensors play an important role in numerous research fields. Quartz crystal microbalances with dissipation monitoring (QCM-Ds) are sensitive devices, and binding events can be observed in real-time. In combination with aptamers, they have great potential for selective and label-free detection of various targets. In this study, an alternative surface functionalization for a QCM-D-based aptasensor was developed, which mimics an artificial cell membrane and thus creates a physiologically close environment for the binding of the target to the sensor. Vesicle spreading was used to form a supported lipid bilayer (SLB) of 1-palmitoyl-2-oleoyl-glycero-3-phosphocholine (POPC) and 1,2-dipalmitoyl-sn-glycero-3-phosphethanolamine-N-(cap biotinyl) (biotin-PE). The SLB was then coated with streptavidin followed by applying a biotinylated aptamer against thrombin. SLB formation was investigated in terms of temperature and composition. Temperatures of 25 °C and below led to incomplete SLB formation, whereas a full bilayer was built at higher temperatures. We observed only a small influence of the content of biotinylated lipids in the mixture on the further binding of streptavidin. The functionalization of the sensor surface with the thrombin aptamer and the subsequent thrombin binding were investigated at different concentrations. The sensor could be reconstituted by incubation with a 5 M urea solution, which resulted in the release of the thrombin from the sensor surface. Thereafter, it was possible to rebind thrombin. Thrombin in spiked samples of human serum was successfully detected. The developed system can be easily applied to other target analytes using the desired aptamers.

## 1. Introduction

Biosensors are of great interest in various research fields, including medicine, biology, environmental control, and food monitoring [1,2,3]. They consist of three parts. The first part is the biological recognition element (1), where a chemical, physical, or biological event occurs. This event is translated by a physical sensor or transducer (2) into a measurable signal, which can then be observed via the signal processor (3). Depending on the type of transducer, different types of biosensors can be distinguished. The most common types are optical, electrochemical, and piezoelectric sensors [4,5,6,7]. The biological recognition element should be sensitive and selective for the target analyte. Many different substances like antibodies, aptamers, and enzymes can be utilized for this purpose [8,9,10]. Sensors that use aptamers are also called aptasensors.

Aptamers are single-stranded RNA or DNA molecules with a length of 20–90 nucleotides. They are selected by a combinatorial chemistry process known as the systematic evolution of ligands by exponential enrichment (SELEX). With their unique three-dimensional (3D) structure, aptamers can bind specifically to a range of targets, from small molecules to proteins to entire cells. The first aptamers were synthesized in 1990 by Turk and Gold, as well as by Ellington and Szostak [11,12]. Compared with antibodies, aptamers provide better thermal and chemical stability. They can be easily modified and synthesized in vitro at a lower cost. Contrary to antibodies, aptamers can be regenerated, making them reusable. With these characteristics, aptamers are ideal candidates for biosensing elements [13].

This work focuses on an aptasensor based on a quartz crystal microbalance (QCM-D). The QCM-D uses a piezoelectric quartz crystal as a transducer. When an alternating voltage is applied to a piezoelectric crystal, it oscillates at its resonant frequency. QCM-D devices have high sensitivity, and limits of detection in the range of nM have been reported [14]. Changes in frequency can be observed in real-time, making them an ideal platform for biosensing. There are already numerous examples of the use of QCM-D-based aptasensors, particularly in the medical field. Aptasensors are being developed for the detection of bacteria, viruses, toxins, and biomarkers [15,16].

For the use of the QCM-D system as a biosensor, various methods are available to functionalize the surface of a quartz crystal with the desired recognition element, mostly based on the strong interaction of thiols with the gold surface of the electrode [15,17,18]. However, it is well known that the absorption of proteins on solid surfaces can lead to a conformational change in the molecules, with possibly reduced activity. Therefore, a chemical environment that does not interfere with the investigated process is favorable. There are several approaches to mimicking biological interfaces, especially cell membrane-like structures, reported in the literature [19,20,21]. They include large and small unilamellar vesicles (LUVs and SUVs, respectively), supported lipid bilayers (SLBs), or black lipid membranes (BLMs) [19,22].

Thrombin plays a crucial role in blood coagulation. The initiation of coagulation activation leads to the proteolytic cleavage of prothrombin to thrombin. The generated thrombin acts as a serine protease, converting soluble fibrinogen into insoluble fibrin and catalyzing several coagulation-related reactions [23,24]. Disruption of the thrombin balance can result in various diseases, ranging from thrombosis to neurodegenerative diseases and cancer [25,26,27]. Therefore, the detection and monitoring of thrombin levels in the blood plays an important role in medical diagnosis. In recent years, much research has been conducted in the field of thrombin biosensor developments [28,29,30]. Several aptamers that bind to thrombin have been described and combined with biosensing systems [31,32,33,34].

Here, we describe a method for immobilization that utilizes a supported lipid bilayer (SLB). SLBs are well-established model systems which mimic the characteristics of a cell membrane. The most interesting properties of these artificial membranes for biosensing are the maintenance of two-dimensional fluidity and the resulting self-organization of the molecules involved [35,36]. SLBs composed of phospholipids are already being used in biosensing technologies [37]. These artificial bilayers can be further modified with streptavidin. Therefore, the headgroups of the used phospholipids have to be partially functionalized with biotin [38]. After streptavidin is attached to the bilayer, a recognition element conjugated with biotin can bind to the streptavidin. When combined with temperature control and buffer composition, an SLB-coated sensor provides a surface which mimics physiological conditions.

In this study, we present a label-free approach for detecting thrombin using an aptamer-based QCM-D biosensor mimicking physiological conditions, in terms of surface functionalization and temperature. Therefore, the quartz surface was functionalized with an SLB composed of a phospholipid mixture. The formation of the bilayer was first examined regarding the temperature and mixing ratio of the used lipids. After optimization, a thrombin-specific aptamer was immobilized on the SLB. Moreover, the sensor was successfully reconstituted for repeated thrombin binding. The high selectivity of the aptamer enabled measurements even in complex matrices such as human serum. With the QCM-D system, binding events can be observed and directly evaluated in real-time.

## 2. Materials and Methods

### 2.1. Reagents

First, 1-palmitoyl-2-oleoyl-glycero-3-phosphocholine (POPC) and 1,2-dipalmitoyl-sn-glycero-3-phosphoethanolamine-N- (cap biotinyl) (sodium salt) (biotin-PE) were obtained from Avanti Polar Lipids (Birmingham, Alabama, USA). Bovine serum albumin (BSA) and phosphate buffer (PBS) containing CaCl_2_ and MgCl_2_ were purchased from Sigma-Aldrich (Taufkirchen, Germany). Streptavidin was obtained from VWR International (Darmstadt, Germany). Thrombin from human serum was bought from Merck (Darmstadt, Germany). Biotinylated thrombin aptamer NU172 (Biotin-5′-CGCCTAGGTTGGGTAGGGTGGTGGCG-3′) was ordered from Ella Biotech (Fürstenfeldbruck, Germany). Tris buffer containing 0.01 M TRIS, 0.1 M NaCl, and 0.01 M CaCl_2_ was adjusted to a pH level of 8. The urea for the 5 M solution was obtained from Fluka Honeywell (Charlotte, NC, USA). The alkaline SDS solution contained 2% SDS, 30 mM EDTA, and 1 M NaOH. Before use, the solution was sonicated until it was clear.

### 2.2. QCM-D Measurements

Quartz crystal microbalance with dissipation monitoring (QCM-D) measurements were conducted using a QCell T Q2 system in conjunction with a peristaltic pump (3T analytik, Tuttlingen, Germany). The silicon dioxide-coated 10 MHz sensor crystals were purchased from 3T analytik. Before use, the sensors were treated for 10 min with an alkaline SDS solution. Subsequently, the sensors were washed with Milli Q water and ethanol and dried under a nitrogen stream. Afterward, the sensors were treated with plasma for 5 min at 100 W using a Zepto HA kHz system (Diener electronics, Ebhausen, Germany). 

The buffer and samples were applied to the sensor at a flow rate of 75 µL*min^−1^ using a peristaltic pump (3T analytik). The buffers were degassed by sonication. All solutions were tempered to 37 °C before use unless stated otherwise. The temperature of the QCM-D was set to 37 °C unless stated otherwise. The frequency Δf and damping ΔΓ were measured in Hz.

### 2.3. Sample Preparation

To prepare small unilamellar vesicles, lipids were dissolved in a 1:1 mixture of methanol and chloroform at a concentration of 5 mg/mL. Appropriate amounts of lipids were mixed in biotin-PE/POPC ratios of 2.5/97.5, 5/95, 10/90, and 20/80. The solvent was evaporated using a nitrogen stream and dried under a vacuum for 2 h. The obtained lipid film was dissolved in Tris buffer, and the lipid solution was subjected to five freeze-thaw cycles and then extruded 21 times through a membrane with a pore size of 50 nm using a mini-extruder (Avanti Polar Lipids). Afterward, the vesicle solution was diluted with Tris buffer to a concentration of 1 mg/mL.

Streptavidin was dissolved in PBS at a concentration of 100 µg/mL. BSA was dissolved in PBS at a concentration of 1 mg/mL. Aptamer NU172 was dissolved in PBS containing 0.1% BSA at concentrations of 0.05, 0.1, 0.25, 0.5, and 0.75 µM. Before use, the aptamer NU172 solutions were heated to 95 °C for 5 min and cooled to room temperature (RT). Thrombin was dissolved in PBS containing 0.1% BSA at concentrations of 1, 2.5, 4, 5, 7, 10, 15, and 25 µg/mL. All buffers and reagents were tempered to 37 °C in a water bath before use, and the buffers were degassed in an ultrasonic bath.

### 2.4. Immobilization of Aptamers and Analysis of Thrombin Binding

**Figure 1:**
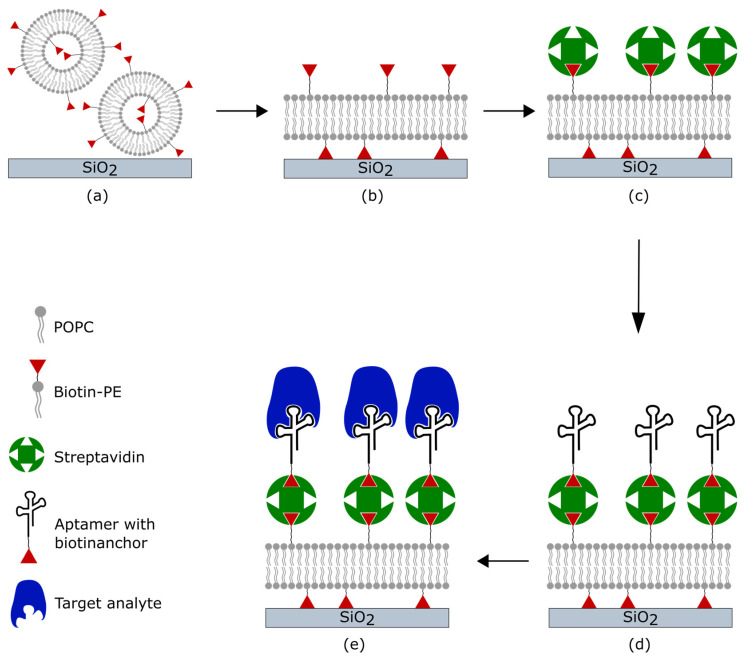
Schematic of the coating procedure and binding of the target analyte. (**a**) Adsorption of phospholipid vesicles on the silicon dioxide surface of the quartz. (**b**) After a final concentration is reached, the vesicles rupture and spread to form an SLB. (**c**) Streptavidin binds to the biotin-modified phospholipids. (**d**) Aptamers functionalized with biotin tethering to streptavidin. (**e**) The target analyte is bound by specific aptamers.

Prior to the quartz crystal surface coating, a baseline was established with Tris buffer. The first step in the immobilization process involved applying vesicles, which resulted in the formation of an SLB. For this purpose, the vesicle solution was applied until a stable signal was achieved. Afterward, the measurement chamber was washed with PBS for 10 min. Subsequently, 300 µL of a 0.1 mg/mL streptavidin solution was applied and allowed to react with the SLB for 10 min. During these 10 min, the pump was stopped. To minimize non-specific interactions, a BSA solution of 1 mg/mL was applied under a constant flow for 5 min and then rinsed with PBS for 5 min. Following this, 250 µL of aptamer solution was added and allowed to react with the previous layer of streptavidin for 10 min. During this time, the pump was stopped once again. Afterward, thrombin solutions were applied and allowed to react for 10 min with the pump turned off. Between the addition of the different compounds to the layers, the chamber was rinsed with PBS for 10 min unless otherwise indicated. All binding events were observed in real-time. In Figure 1, a schematic representation of the immobilization of the aptamer is shown.

### 2.5. Determination of the Limit of Detection (LOD)

The concentration of thrombin was varied between 1 and 25 µg/mL to establish a calibration curve. The linear regression analysis between the frequency shift and thrombin concentration was used to calculate the LOD according to Equation (1):

(1) $$LOD=\frac{3.3\cdot \sigma_{K}}{S}$$

where $\sigma_{K}$ represents the standard deviation of the intercept and S is the slope of the regression line.

### 2.6. Reconstitution of the Sensor

To test the reconstitution of the sensor, the sensor was washed with a 5 M urea solution to release bound thrombin from the aptamers on the sensor’s surface. After the frequency had stabilized, a washing step with PBS containing 0.1% BSA was performed, and thrombin was applied for a second time. During the removal of thrombin from the sensor, the samples were collected and concentrated using an Amicon filter (10K MWCO) (Merck, NJ, USA). The released thrombin was determined by thrombin ELISA (Abcam, Cambridge, UK) according to the manufacturer’s instructions. The samples were diluted at a 1:1000 ratio, and the absorbance was measured at 450 nm with the correction wavelength set at 540 nm using a microplate reader (Eon Synergy 2, BioTek Instruments, Winooski, VT, USA). All experiments were repeated three times, and standard deviations (SDs) were calculated.

## 3. Results and Discussion

Thus far, the formation of bilayers through vesicle fusion on SiO_2_ quartz crystals has been investigated with respect to the size of the vesicles, as well as the pH and ionic strength of the buffer used [35,36]. Bilayers themselves are often investigated at RT. However, there is rather little research on the influence of temperature on SLB formation, mostly focusing on the SLB formation of 1,2-dioleoyl-sn-glycero-3-phosphocholine (DOPC) below or above its phase transition temperature (T_m_) [39,40,41]. Since our goal was to conduct the measurements at 37 °C to mimic physiological conditions, bilayer formation was investigated between 15 and 40 °C. Furthermore, we also examined how the composition of the used lipids affected the formation of the bilayer.

### 3.1. Influence of Temperature on SLB Formation

**Figure 2:**
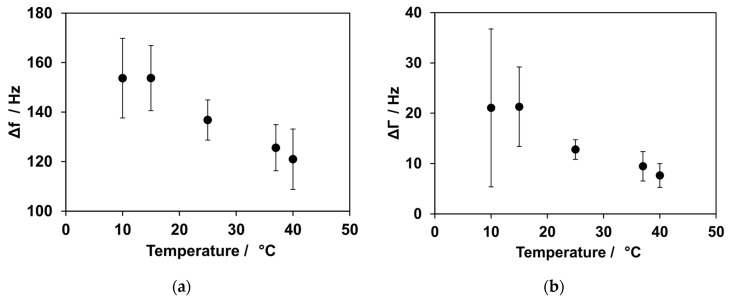
Temperature dependence for SLB formation. (**a**) The influence of temperature on frequency shifts. At low temperatures, an increased shift in frequency was observed, while the shift was smaller at higher temperatures. (**b**) Influence of temperature on damping. At low temperatures, a higher shift in damping could be observed compared with higher temperatures.

During SLB formation, the intact vesicles initially adsorb onto the SiO_2_ surface of the quartz. This leads to an increase in mass and results in a decrease in frequency. When a critical concentration of vesicles is reached, the vesicles rupture and spread, leading to the formation of an SLB. The rupture of vesicles depends on both their concentration on the surface and their size. Compared with large vesicles, vesicles with a small diameter experience greater stress to deformation caused by surface interaction and interaction between the vesicles themselves [40]. This vesicle rupture is accompanied by an increase in frequency. The formation of an SLB was measured at 10, 15, 25, 37, and 40 °C. The results are shown in Figure 2. The SLB formation was slower at low temperatures compared with high temperatures. At 15 °C, SLB formation took approximately 23 min, whereas at 40 °C, the formation was complete after 11 min. The resulting frequency shift varied at the temperatures investigated. Frequency shifts of 121 ± 12 Hz and 126 ± 9 Hz were observed for 40 °C and 37 °C, respectively. A much higher frequency shift was detected at lower temperatures: 155 ± 20 Hz at 10 °C, 153 ± 13 Hz at 15 °C and 136 ± 8 Hz at 25 °C. Higher damping values were observed at lower temperatures. For 10 °C, 21 ± 16 Hz was the result, and for 15 °C, 21 ± 8 Hz was measured. At RT and above, ΔΓ values of 13 ± 2 Hz, 9 ± 3 Hz, and 8 ± 2 Hz were obtained at 25 °C, 37 °C, and 40 °C, respectively.

We observed that the kinetics of bilayer formation are dependent on the temperature. Low temperatures resulted in a slower formation of the bilayer, while high temperatures accelerated its formation. For complete bilayer formation, a consistent drop in frequency should be expected at every temperature. However, there was a much higher decrease in frequency for the low temperatures compared with the high temperatures. This could be explained by an incomplete rupture and spreading of the vesicles on the quartz surface. This means that there were still intact vesicles present on the surface at lower temperatures. Previous studies have investigated the influence of temperature on vesicle fusion with regard to the T_m_ of the used lipids [42]. In their findings, at low temperatures, a higher concentration of vesicles was required to initiate vesicle fusion. Additionally, some of the vesicles remained intact instead of spreading to an SLB. Jing et al. showed that 1,2-dipalmitoyl-sn-glycero-3-phosphocholine (DPPC) vesicles did not rupture spontaneously when the temperature was below their T_m_ of 41 °C [40]. The vesicles used here consisted of a mixture of two different lipids. The T_m_ for POPC and biotin-PE are −2 °C and 32 °C, respectively [43].

The T_m_ for POPC is below the temperatures investigated in this study, and therefore, it did not play a role in further interpretations. The T_m_ for biotin-PE is 32 °C, which falls within the temperature range that was studied. Biotin-PE may inhibit vesicle rupturing at temperatures below 32 °C. Since the vesicles consisted of a mixture of two lipids, the rupturing of vesicles was only partially inhibited.

This theory is in accordance with the effect observed in dampening. The values of ΔΓ were high and showed a large standard deviation at low temperatures. This can be a sign of intact vesicles on the quartz surface. The vesicles were filled with buffer, which resulted in high damping and an increase in mass when they came into contact with the quartz. Both of these effects were observed during measurements and became less noticeable with increasing temperatures.

### 3.2. Influence of Percentage of Biotinylated Phospholipids on Bilayer Formation and Binding of Streptavidin

The influence of temperature on bilayer formation was investigated using a lipid mixture consisting of 95% POPC and 5% biotin-PE. To bind additional layers, the concentration of biotinylated lipids had to be optimized. These experiments were conducted at a temperature of 37 °C. To achieve optimal binding of streptavidin to the bilayer, the content of biotin-PE was varied between 2.5% and 20%. After the bilayer formation of the lipid mixes containing varying percentages of biotin-PE, the binding of streptavidin followed.

The kinetics of bilayer formation varied depending on the concentration of biotin-PE in the mixture. The frequency shifts for SLB formation were similar for biotin-PE concentrations of 2.5%, 5%, and 10%, and the mean values varied between 98 Hz and 120 Hz. With a high biotin-PE content of 20% in the lipid mixture, the frequency shift was higher, with values of approximately 168 ± 46 Hz.

**Figure 3:**
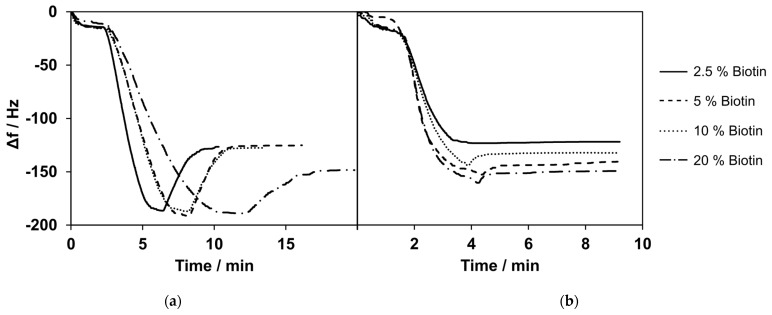
(**a**) Selected measurements of bilayer formation with varying percentages of biotinylated phospholipids. Here, 20% biotinylated phospholipids showed a higher change in frequency compared with the lower percentages of biotinylated phospholipids. (**b**) Selected measurements of streptavidin (c = 100 µg/mL) binding to bilayers composed of lipid mixtures containing 2.5–20% biotinylated phospholipids. Lipid mixes with a lower percentage of biotinylated phospholipids showed a smaller frequency change upon the addition of streptavidin. Selected measurements are presented. The curves were normalized to 0 Hz. All experiments were conducted at 37 °C.

After the bilayer formation with varying concentrations of biotin-PE, 100 µg/mL of streptavidin was added. Figure 3 shows the exemplary measurements for the formation of lipid bilayers with varying contents of biotin (a) and the subsequent binding of streptavidin (b). The frequency shift for the binding of streptavidin was similar for biotin-PE concentrations of 2.5%, 5%, and 10%, with values of 123 ± 2 Hz, 138 ± 4 Hz, and 138 ± 8 Hz, respectively. With a higher biotin-PE concentration in the bilayer, a larger frequency shift was observed when streptavidin was added. The shift was 166 ± 17 Hz for 20% biotin-PE content in the SLB.

The frequency shift values for SLB formation and the subsequent binding of streptavidin did not differ much for the mixes containing up to 10% of biotinylated lipids. For 20% biotinylated lipids, the formation of SLBs showed a higher change in frequency and higher standard deviations. The difference between 5% and 10% biotinylated lipids was minimal for SLB formation as well as streptavidin binding. Therefore, we decided to use a content of 5% biotinylated lipids for all further experiments, in accordance with the previous literature [44,45].

### 3.3. Immobilization of Aptamer

**Figure 4:**
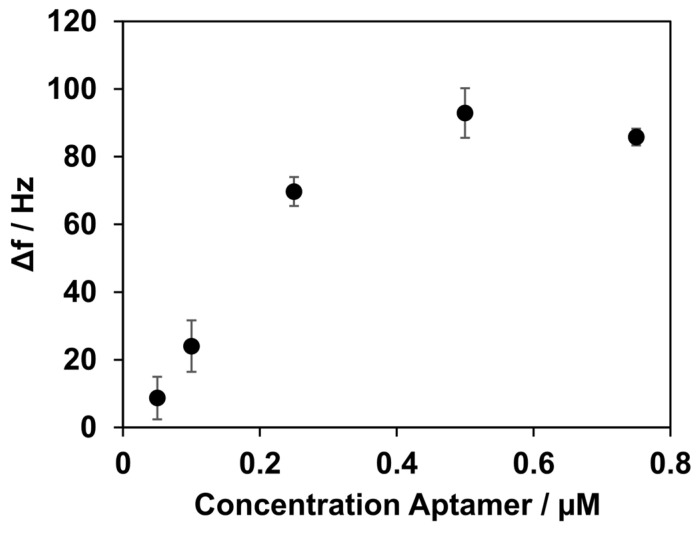
Frequency shifts after the immobilization of aptamer NU172, depending on its concentration. Concentrations of 0.05, 0.1, 0.25, 0.5, and 0.75 µM were measured. Increasing the concentration of aptamer NU172 to up to 0.5 µM resulted in an increase in Δf. Higher changes in Δf were not observed when a higher concentration was applied. All experiments were conducted at 37 °C.

To ensure sufficient binding of aptamer NU172 to the coating of the lipid bilayer and streptavidin, various concentrations of the aptamer were tested. Rising frequency shifts were observed for concentrations of 0.05 µM, 0.1 µM, and 0.25 µM, with values of 9 ± 6 Hz, 24 ± 8 Hz, and 70 ± 4 Hz, respectively. The highest frequency shift was observed at a concentration of 0.5 µM, with 93 ± 7 Hz. A further increase in aptamer NU172’s concentration to 0.75 µM did not result in a higher frequency shift, as shown in Figure 4. The frequency shift for the concentration of 0.75 µM was 86 ± 3 Hz.

These results suggest a favourable concentration for binding aptamer NU172 of 0.5 µM. With concentrations below 0.5 µM, the binding capacity of the lipid bilayer seemed to not be fully utilized. A concentration of aptamer NU172 above 0.5 µM did not result in a greater decrease in frequency. This observation led to the conclusion that no further binding of the aptamer was possible, and saturation was reached. Nevertheless, this does not necessarily imply that all aptamers are available for binding of the analyte. In general, steric hindrance can occur, depending on the size of the target, such that one-to-one occupancy of the aptamer layer is not automatically given. For the following experiments, a final concentration for aptamer NU172 of 0.5 µM was used.

### 3.4. Binding of Thrombin and Determination of the LOD

**Figure 5:**
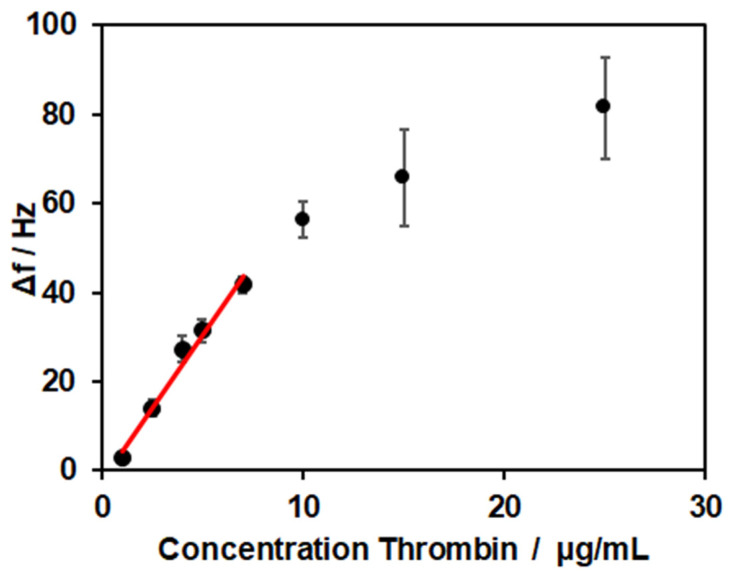
Frequency shifts for thrombin binding in concentrations of 1, 2.5, 4, 5, 7, 10, 15, and 25 µg/mL. All experiments were conducted at 37 °C.

After establishing a coating based on the lipid bilayer, streptavidin, and the thrombin-specific aptamer NU172, the next step was analysis of the thrombin binding. Different concentrations of thrombin ranging from 1 µg/mL to 25 µg/mL were tested (Figure 5). For the concentration of 1 µg/mL, a frequency shift of 3 ± 1 Hz was observed. The shift increased with higher concentrations of 2.5, 4, 5, 7, 10, 15, and 25 µg/mL, resulting in frequency shifts of 14 ± 2 Hz, 27 ± 3 Hz, 31 ± 3 Hz, 42 ± 2 Hz, 56 ± 4 Hz, 66 ± 11 Hz, and 81 ± 11 Hz, respectively. The curve showed a linear relationship in the range of 1–7 µg/mL for the thrombin concentration (R^2^ = 0.9814). For higher concentrations, saturation of the sensor with a reduced binding capacity for thrombin occurred.

The limit of detection was calculated from the linear range of detection (27–189 nM) to LOD = 1 µg/mL, which corresponded to a concentration of 29 nM. In the literature, values of 0.1–1 nM for QCM-based thrombin sensors were reported [46,47,48]. Here, the signal of thrombin binding was enhanced by inorganic structures like magnetic beads or gold nanoparticles. In the work of Hianik et al. [49], the sensor’s surface was modified with 3,3′-dithiopropionic acid-di(N-succinimidylester) (DSP), and the biotinylated aptamer was immobilized via biotin–avidin interaction. With this set-up, they reached a limit of detection of 1 nM. To examine if the surface modification with the SLB was responsible for the low sensitivity observed, experiments with a biotinylated PEG thiol, forming a self-assembled monolayer, and thrombin concentrations of 2.5 and 5 µg/mL were conducted. The resulting frequency shifts of 13 ± 2 Hz and 37 ± 3 Hz showed no clear differences compared to the values obtained for the SLB-modified sensors. Therefore, no direct influence of surface modification on the LOD was revealed.

### 3.5. Removal of Thrombin

To analyse the possibility of reconstitution of the sensor, a method to release thrombin from the aptamer NU172 was explored. The detachment of thrombin can be achieved by denaturing the 3D structure of the aptamer, as the specific 3D structure is crucial for binding to the target analyte. Urea can destabilize and denature the native conformation of nucleic acids by acting as a hydrogen bond donor and acceptor [50,51].

**Figure 6:**
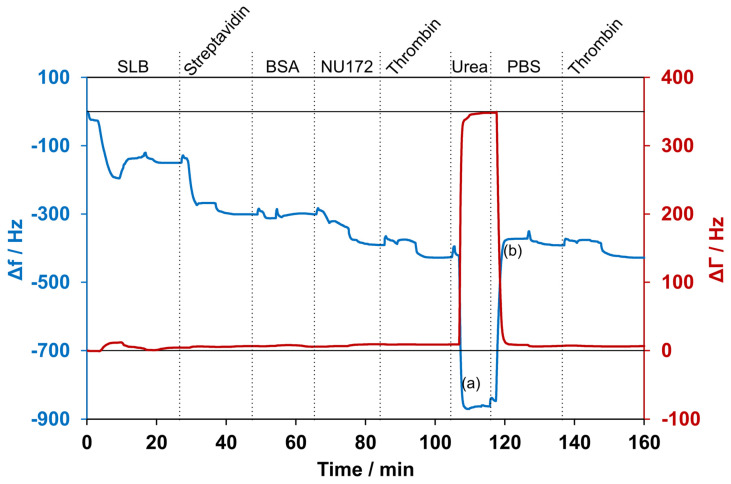
Selected measurement with detection of thrombin and subsequent removal using a 5 M urea solution. The blue line shows the changes in frequency, while the red line shows the changes in damping. (a) and (b) indicate the times at which samples for ELISA were collected. First, a supported lipid bilayer (SLB) was formed and incubated with streptavidin. After washing with 1 mg/mL BSA solution, aptamer NU172 was added. Subsequently, the sensor was exposed for the first time to thrombin. Then, the washing with urea resulted in a high decrease in frequency and an increase in damping. After rinsing with PBS, the frequency increased, and the damping decreased again. The application of thrombin for a second time resulted in a decrease in frequency. The experiment was conducted at 37 °C, and curves were normalized to 0 Hz.

After the generation of the aptamer coating, thrombin incubation resulted in a frequency drop of 50 ± 11 Hz. When the urea solution was applied to the system, a sharp frequency drop of 454 ± 28 Hz was observed. At the same time, the damping increased by 316 ± 110 Hz. After rinsing with PBS, a frequency increase of 489 ± 26 Hz was observed, which stabilized at approximately the same frequency monitored before the addition of thrombin. The huge change in frequency and damping during washing with urea was probably due to the different properties of the 5 M urea solution, like the viscosity and density, compared with the PBS, as well as interactions with all layers of the system. To demonstrate that the aptamer remained active and refolded after the removal of urea, thrombin was applied for a second time to the reconstituted sensor surface. The repeated thrombin application resulted again in a frequency decrease of 42 ± 8 Hz and was comparable to the decrease seen the first time thrombin was applied. One representative measurement is depicted in Figure 6. Furthermore, multiple cycles of thrombin application and urea-induced detachment were performed. A steady decrease in the signal over six cycles was observed: 63 ± 6 Hz, 54 ± 0.3 Hz, 73 ± 17 Hz, 41 ± 1 Hz, 38 ± 1 Hz, and 31 ± 3 Hz, respectively. The frequency decrease from the second to fifth additions of thrombin was in the range of the generally observed fluctuations.

**Figure 7:**
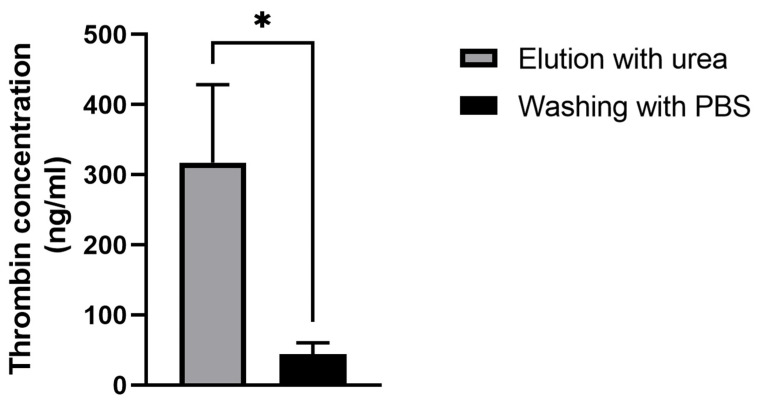
Detection of thrombin in the eluates after incubation with the 5 M urea solution (a) and the following washing with PBS (b) using ELISA. The results are shown as mean + SD (n = 3). Statistical differences were determined using an unpaired *t*-test (* *p* < 0.05).

To detect thrombin detached from the aptamer NU172 on the sensor’s surface, ELISA was conducted with two samples collected during the QCM-D experiment. The first eluate was collected while washing with urea (1), and the second eluate was collected during the rinsing with PBS after urea incubation (2). As shown in Figure 7, thrombin was successfully removed from the sensor’s surface with the urea treatment. Increased thrombin amounts were detected in the urea solution (317.2 ± 111.3 ng/mL) compared with the PBS washing solution (43.76 ± 16.94 ng/mL).

These findings led to the conclusion that it is possible to successfully remove thrombin from its specific aptamer and reuse the sensor for a second time. This improves the sensor system presented in terms of both resource utilization and time spent on preparation.

### 3.6. Thrombin Detection in Human Serum

After the successful binding of thrombin in a defined buffer solution, the performance of the established sensor was tested in a more complex matrix. Therefore, human serum with a 1:500 dilution in PBS containing 0.1% BSA was spiked with thrombin. In addition, control measurements were conducted using samples that contained only the serum without the addition of thrombin.

**Figure 8:**
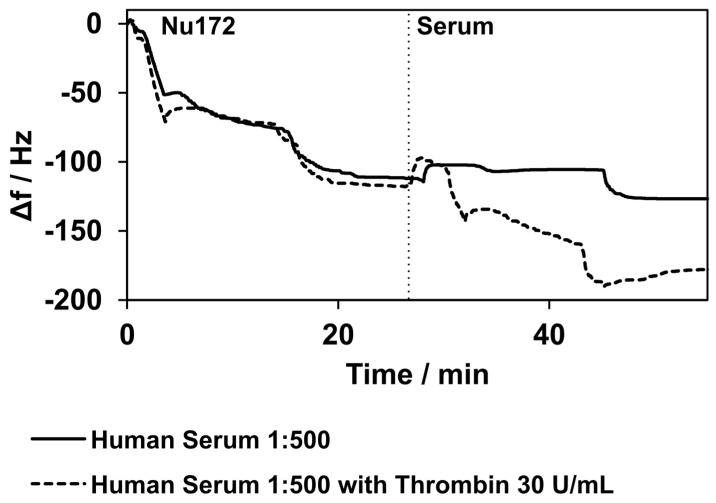
Binding of aptamer NU172 and binding to thrombin in human serum diluted at a 1:500 ratio with PBS. Two selected measurements are presented. The solid line shows the measurements with pure human serum, while the dashed line represents the measurements with human serum spiked with thrombin. The curves match during the immobilization of the aptamer NU172. When the human serum was applied, a slight decrease in frequency was observed, but the decrease in frequency was considerably higher when the human serum was spiked with thrombin. The curves were normalized to 0 Hz at the point of aptamer NU172’s application. Experiments were conducted at 37 °C.

A frequency decrease of 11 ± 19 Hz was observed when the human serum without thrombin’s addition was applied. The samples spiked with thrombin showed a frequency decrease of 61 ± 10 Hz. In Figure 8, the example measurements show a direct comparison between the pure serum and the spiked serum. No changes in damping were observed for either the pure serum or the spiked serum.

## 4. Conclusions

In this study, an alternative surface functionalization for a sensor system based on QCM-D and aptamers was developed to detect targets under an almost physiological environment. The coating of the quartz crystal was formed by creating an SLB that served as an artificial membrane maintaining two-dimensional fluidity, which allowed self-organization of the molecules involved. In the next step, streptavidin was added, which acted as an anchor for binding of the biotinylated thrombin-specific aptamer.

SLB formation at temperatures of 25 °C and below was partially inhibited due to the T_m_ of biotin-PE. Regarding the content of biotinylated lipids in the mixture, the differences in SLB formation and the subsequent binding of streptavidin were minor for contents ranging from 2.5 to 10% biotin-PE. The binding of aptamer NU172 and thrombin was optimized in terms of applied concentrations. In both cases, saturation commenced at certain concentrations. Thrombin was detected both in the buffer as well as in a complex matrix of human serum. Furthermore, the captured thrombin could be released through a washing step with urea, which enabled the reconstitution of the sensor.

Functionalization of the sensor surface with an SLB provides the advantage of a cell membrane-like interface. This offers the possibility to study biological binding events in a chemical environment that is closer to physiological conditions and understand processes at the molecular level. Most biosensor systems aim to detect a target with high sensitivity. The aptamer-based QCM-D biosensor presented in this work had the objective to create a platform for real-time analysis of biological interactions like allergen–antigen or aptamer binding or cell-based studies. The limit of detection of 29 nM offers a sufficient range for studies focusing on mechanistic questions.

The sensor design can be applied to any target analyte as long as a suitable aptamer is available. The label-free approach and the ability to follow binding events in real-time make this QCM-D-based aptasensor highly promising for various research areas where deeper insights into the nature of binding processes are required.

## Data Availability

Dataset available on request from the authors.
